# Artificial intelligence-based nomogram for small-incision lenticule extraction

**DOI:** 10.1186/s12938-021-00867-7

**Published:** 2021-04-23

**Authors:** Seungbin Park, Hannah Kim, Laehyun Kim, Jin-kuk Kim, In Sik Lee, Ik Hee Ryu, Youngjun Kim

**Affiliations:** 1 Center for Bionics, Korea Institute of Science and Technology, Seoul, Korea; 2 Division of Bio-Medical Science &Technology, KIST School, Korea University of Science and Technology, Seoul, Korea; 3 B&VIIT Eye Center, Seoul, Korea

**Keywords:** SMILE, Nomogram, Artificial intelligence, Machine learning

## Abstract

**Background:**

Small-incision lenticule extraction (SMILE) is a surgical procedure for the refractive correction of myopia and astigmatism, which has been reported as safe and effective. However, over- and under-correction still occur after SMILE. The necessity of nomograms is emphasized to achieve optimal refractive results. Ophthalmologists diagnose nomograms by analyzing the preoperative refractive data with their individual knowledge which they accumulate over years of experience. Our aim was to predict the nomograms of sphere, cylinder, and astigmatism axis for SMILE accurately by applying machine learning algorithm.

**Methods:**

We retrospectively analyzed the data of 3,034 eyes composed of four categorical features and 28 numerical features selected from 46 features. The multiple linear regression, decision tree, AdaBoost, XGBoost, and multi-layer perceptron were employed in developing the nomogram models for sphere, cylinder, and astigmatism axis. The scores of the root-mean-square error (RMSE) and accuracy were evaluated and compared. Subsequently, the feature importance of the best models was calculated.

**Results:**

AdaBoost achieved the highest performance with RMSE of 0.1378, 0.1166, and 5.17 for the sphere, cylinder, and astigmatism axis, respectively. The accuracies of which error below 0.25 D for the sphere and cylinder nomograms and 25° for the astigmatism axis nomograms were 0.969, 0.976, and 0.994, respectively. The feature with the highest importance was preoperative manifest refraction for all the cases of nomograms. For the sphere and cylinder nomograms, the following highly important feature was the surgeon.

**Conclusions:**

Among the diverse machine learning algorithms, AdaBoost exhibited the highest performance in the prediction of the sphere, cylinder, and astigmatism axis nomograms for SMILE. The study proved the feasibility of applying artificial intelligence (AI) to nomograms for SMILE. Also, it may enhance the quality of the surgical result of SMILE by providing assistance in nomograms and preventing the misdiagnosis in nomograms.

**Supplementary Information:**

The online version contains supplementary material available at 10.1186/s12938-021-00867-7.

## Background

Small-incision lenticule extraction (SMILE) has been reported as safe and effective for correcting refractive errors [1, 2]. However, over- and under-correction still occur after SMILE [3, 4]. The surgical outcome of a refractive ophthalmic surgery is affected by various factors, such as the surgeon, surgical process, type of laser used, patient demographics, and operation room environment [5]. The necessity of nomograms is emphasized to compensate for these sources of variability and achieve optimal refractive results [6]. Nomograms are considered as reliable and efficient tools for improving the predictability of a refractive surgery by analyzing the preoperative and postoperative refractive data [6, 7].

Numerous studies suggesting nomograms for laser-assisted in situ keratomileusis (LASIK) and SMILE have been conducted [5, 7–11]. Most previous studies focused only on the amount of the spherical or cylindrical refraction power to correct, excluding the astigmatism axis despite its influence on astigmatism and visual acuity after LASIK. [12]. Furthermore, the linear regression analysis was generally used to select significant parameters highly related to the postoperative results and develop an equation in most of the previous studies. In addition, the nomogram development for SMILE has not been broadly studied yet.

Applying artificial intelligence (AI) in medical fields has become mainstream with the digital clinical data storage expansion and related technology advances [13]. In ophthalmology, AI has been applied intensively to diagnose ophthalmological diseases, such as diabetic retinopathy, glaucoma, age-related macular degeneration, and cataract [14]. For nomograms of refractive surgery, a neural network was used to suggest the surgical laser parameter for photorefractive keratectomy [15]. Recently, Tong et al. [16] applied the multi-layer perceptron (MLP) algorithm to train nomogram models for SMILE. However, there was no comparison with the other algorithms in their study.

In this study, an AI-based approach to develop nomograms for SMILE is proposed. Various machine learning algorithms were employed: multiple linear regression, decision tree, AdaBoost, XGBoost, and MLP. Furthermore, the feature importance was calculated, which numerically expresses the effect of specific features on the nomogram decision. To the best of our knowledge, this study is the first to apply diverse machine learning algorithms extensively, other than linear regression or MLP solely, to nomograms for SMILE.

## Results

**Fig. 1:**
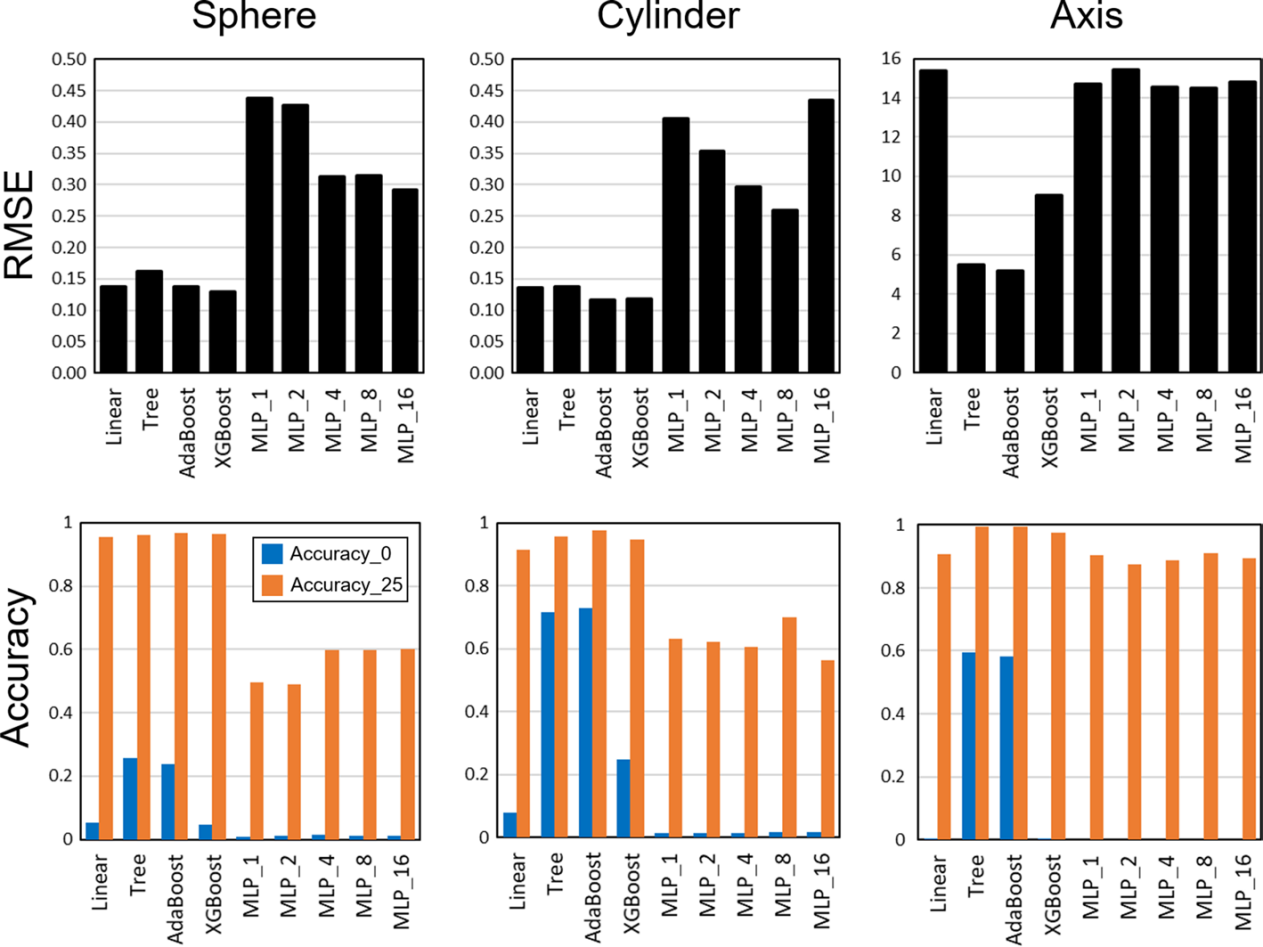
Results of RMSEs and accuracies of multiple linear regression (linear), decision tree (tree), AdaBoost, XGBoost, and MLP with hidden layer number of 1 (MLP_1), 2 (MLP_2), 4 (MLP_4), 8 (MLP_8), and 16 (MLP_16). Axis: astigmatism axis; Accuracy_0: accuracy with threshold of zero; Accuracy_25: accuracy with threshold of 0.25 D for sphere and cylinder and 25° for astigmatism axis nomograms

**Fig. 2:**
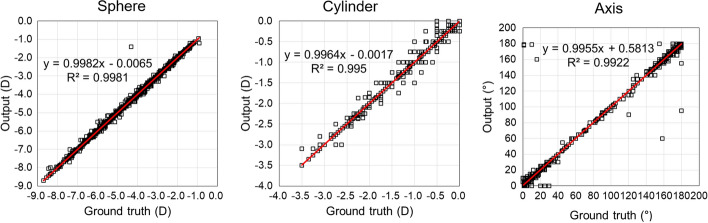
Ground truths vs. outputs from the best model of AdaBoost. Axis: astigmatism axis

Figure 1 displays the root-mean-square errors (RMSEs) and accuracies of all the algorithms for the nomograms of sphere, cylinder, and astigmatism axis. The results indicate that AdaBoost achieved the highest performance with RMSEs of 0.1378, 0.1166, and 5.17 for the sphere, cylinder, and astigmatism axis nomograms, respectively. The corresponding accuracies with a threshold of zero were 0.236, 0.728, and 0.583 for the sphere, cylinder, and astigmatism axis nomograms, respectively. The accuracies with a threshold of 0.25 D for the sphere and cylinder nomograms and 25° for the astigmatism axis nomograms were 0.969, 0.976, and 0.994, respectively. The secondary best algorithm was the decision tree, for which the RMSEs were 0.1622, 0.1376, and 5.47 for the sphere, cylinder, and astigmatism axis nomograms, respectively. The accuracies with a threshold of zero were 0.257, 0.717, and 0.596 for the sphere, cylinder, and astigmatism axis nomograms, respectively. The accuracies with a threshold of 0.25 D for the sphere and cylinder nomograms and 25° for the astigmatism axis nomograms were 0.962, 0.958, and 0.993, respectively. The results of the Mann–Whitney *U* tests display that the ground truths and the outputs from the best models of AdaBoost were not significantly different (*p* < 0.05) for all the sphere, cylinder, and astigmatism axis cases. The correlation of the ground truths and the model outputs is illustrated in Fig. 2.

**Fig. 3:**
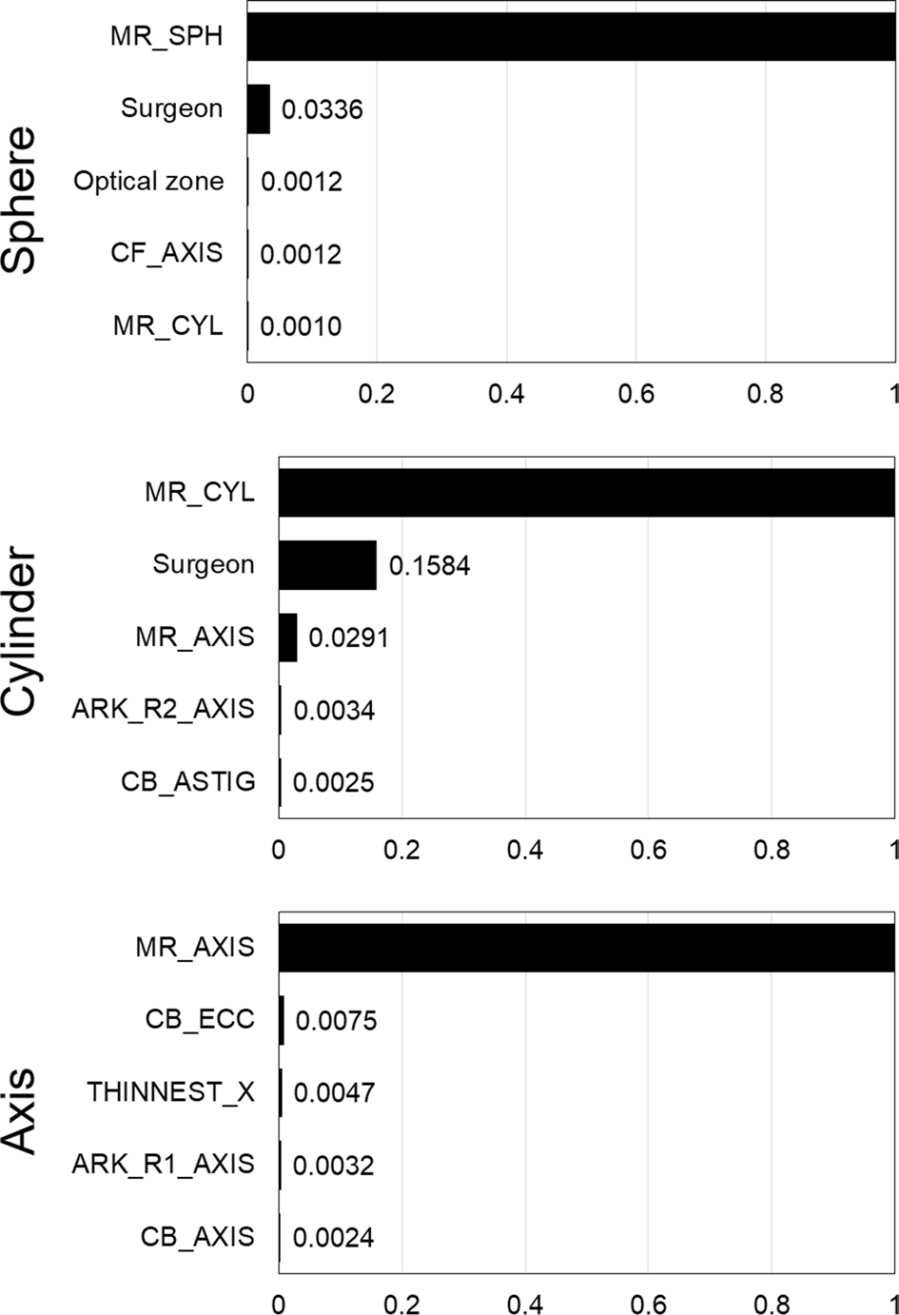
Five features with highly ranked feature importance of best model of AdaBoost for the sphere, cylinder, and astigmatism axis nomograms. *Axis* astigmatism axis, *MR_SPH* manifest refraction of sphere, *CF_AXIS* corneal front astigmatism axis, *MR_CYL* manifest refraction of cylinder, *MR_AXIS* manifest refraction of astigmatism axis, *ARK_R2_AXIS* axis of the steepest curvature, *CB_ASTIG* corneal back astigmatism, *CB_ECC* corneal back eccentricity, *THINNEST_X* x location at thinnest cornea, *ARK_R1_AXIS* axis of flattest curvature, *CB_AXIS* corneal back astigmatism axis

Figure 3 presents five features with a highly ranked feature importance of the best models of AdaBoost for the sphere, cylinder, and astigmatism axis nomograms. The most important feature with a significantly high importance was the preoperative manifest refraction for all the sphere, cylinder, and astigmatism axis cases. For the sphere and cylinder nomograms, the surgeon was the following highest important feature.

## Discussion

To predict the nomograms for SMILE, various machine learning algorithms were applied: multiple linear regression, decision tree, AdaBoost, XGBoost, and MLP with different number of layers. The best performance was achieved with AdaBoost. The RMSE from the results of the multiple linear regression of the sphere and cylinder nomograms was similar to those of AdaBoost; however, the accuracy of the former was remarkably lower than AdaBoost. For the astigmatism axis, the multiple linear regression yielded an extensively high RMSE and low accuracy compared to those of AdaBoost. Comparing the decision tree, AdaBoost, and XGBoost, the decision tree exhibited comparable performance to AdaBoost, whereas XGBoost did not. In addition, although deep learning based on MLP has recently exhibited high performance in other studies, it was not the case in this study. Summarizing the results, relatively simpler models, such as decision tree or AdaBoost, worked better compared to other complex and deep models to predict nomograms for SMILE with the data cohort that was used in this study.

Although deep learning is excellent in the domains of computer vision or natural language processing, it has been reported that shallow models, such as gradient-boosted decision trees, exhibit good performance in problems with tabular heterogeneous data [17]. Although deep learning has garnered significant attention over the last years, gradient boosting, such as XGBoost, is one of the most widely used algorithms in Kaggle competitions for applying machine learning to structured tabular data [18, 19]. Several studies have reported higher performance with AdaBoost compared to XGBoost despite the popularity of XGBoost [20–26].

Compared with the previous studies, this study proposes the following contributions. First, it is a novel approach to apply and compare the extensive range of data-driven machine learning algorithms for nomograms subject to SMILE. There has been no particular approach even for nomograms subject to other refractive surgery. Previous studies mainly applied multiple linear regression to nomograms. Although recently there was a research on applying machine learning to nomograms for SMILE [16], it only attempted shallow MLP.

Compared to the other studies, the number of the data used in this study was remarkably large: hundreds versus thousands. This approach was possible, because the center, i.e., the data provider, has endeavored to establish an enormous database considering the impact of big data and data-driven artificial intelligence technologies in the medical field. Moreover, the features considered to affect the nomograms in this study were extensive compared to other studies. Without an ideal criterion to determine the relevance of the factors for the refractive outcomes, these factors were selected based on scientific studies, common sense, and even a feeling [5]. Although a large number of features does not necessarily ensure a better performance of machine learning, we intended not to miss any relevance between the possible features and the nomograms.

Another contribution of this study was the consideration of the “surgeon” feature. In the results of the feature importance of the models, the surgeon feature had the second-highest importance followed by the preoperative manifest refraction. Unlike LASIK, SMILE nomogram mainly depends on the personal experience of the surgeon [16], and it is essential that all the surgeons develop a nomogram to refine their results [27]. It is certain that the effect of a surgeon on a nomogram is strong. To our knowledge, all the previous studies for nomograms subject to refractive surgery only utilized the data cohort of one sole surgeon, which limits the feasibility of a general application. For example, Liang et al. [7] stated that the nomogram used in their study is not available for other surgeons. We believe that our novel approach could be referential for further enhanced nomogram development for SMILE considering surgeon effect.

The limitation of this retrospective study is the absence of clinical validation. It is necessary to clinically verify that the proposed nomogram enhances the predictability for the postoperative surgical outcomes of SMILE. However, the positive clinical results are anticipated considering the results of Cui et al. [16]. They confirmed the comparable safety and predictability in the postoperative results of patients’ group that underwent SMILE with nomograms from the machine learning model, which had no statistically significant difference compared to the surgeon nomograms.

## Conclusions

To predict the sphere, cylinder, and astigmatism axis nomograms for SMILE, we applied various machine learning algorithms: multiple linear regression, decision tree, AdaBoost, XGBoost, and MLP. The best results were achieved with AdaBoost. The preoperative manifest refraction was the highest important feature for all nomogram cases. The second-highest important features for the sphere and cylinder nomograms were the surgeon. We believe that the proposed novel approach can lead to further development of AI-based nomograms for SMILE. It displayed the feasibility of applying AI to nomograms for SMILE. Although there was no clinical verification, we expect positive refractive results.

## Methods

### Data

The data used in this research were provided by B&Viit Eye Center (Seoul, Korea). We retrospectively analyzed the data of 2108 eyes from 1336 patients operated by expert ophthalmologist A and 1059 eyes from 546 patients operated by expert ophthalmologist B between 2014 and 2018. All the patients underwent SMILE. Each one was operated after being anesthetized with 0.5% proparacaine hydrochloride (Alcain®, Alcon, Purrs, Belgium). A Visumax laser (Visumax™ Femtosecond Laser, Carl Zeiss Meditec) was used to cut the lenticule. The spot energy used was 130 nJ with a spot distance of 3 μm and a repetition rate of 500 kHz. The lenticule cut angle and the cut size were 145° and 2.0 mm, respectively. The lenticule diameter was 6.0–6.5 mm, the cap diameter was 7.5 mm, and the cap thickness was 120 μm. When the patients were looking at a frontal green light, a corneal connector was placed in the middle of the cornea, and a contact surface was created through a tear. After the laser irradiation, the superficial and deep planes of the lenticule were dissected using a spatula inserted through the lenticule cut and the lenticule was extracted using forceps. The surgical procedure was finished after washing the intrastromal space with a balanced solution (BSS®, Alcon Laboratories, Inc. Fort Worth, TX, USA). After the surgery, the patients were instructed to apply moxifloxacin (Vigamox®, Alcon, Fort Worth, TX, USA) and Lotemax eye drops (Lotemax®, Bausch + Lomb, Inc., Bridgewater, NJ, USA) three times a day for 2 weeks.

All the subjects underwent preoperative evaluations, which consisted of the manifest refraction test, corrected-distance visual acuity test, measurements of intraocular pressure (NT-530P, NIDEK, Japan), and refraction measurements conducted via automated keratometry (ARK-530A, NIDEK, Japan), Pentacam (Pentacam®, Oculus, Germany), and topography (Topography, Oculus, Germany). The postoperative evaluations included uncorrected-distance visual acuity (UDVA) test by automated keratometry (ARK-530A, NIDEK, Japan) after 3 months from the procedure performed. The data inclusion criteria were postoperative logMAR UDVA of + 0.9 or better with no postoperative traumas. After excluding the data that does not satisfy the inclusion criteria and removing the missing values, the data of 3034 eyes were used.

The data consisted of 46 numeric features and four categorical features: age, gender, right or left of eye, and surgeon. Among 46 numeric features, 28 features that were selected from each cluster among the hierarchical clusters by the Spearman rank-order correlations were used. The categorical features were discretized into integer classes and one-hot encoded for training. The nomograms for the sphere, cylinder, and astigmatism axis determined by expert ophthalmologists A and B served as the target output, i.e., ground truth. The names and the statistical characteristics of all the selected features and the nomograms of the experts are provided in Additional file 1.

### Algorithms

To develop the nomogram models, various machine learning algorithms were employed: multiple linear regression, decision tree algorithm called classification and regression trees (CART) [28], AdaBoost [29], XGBoost [30], and MLP. In CART, a decision tree learns from the given training data by repeating a binary recursive partitioning, eventually building a tree structure. The conditional tests are conducted at the nodes, which are the partitioning points of trees with specific thresholds to achieve the largest variance reduction. The criterion used to split at the nodes was the mean-squared error. The depth of the tree model was seven for the sphere and cylinder and five for the astigmatism axis. The minimum number of samples in a node was set to one.

Boosting is a general method for improving the performance of any learning algorithm by running weak models on various distributions over the training data, and subsequently combining them into a single composite model [31]. In repetitive training process, AdaBoost allows model to focus on the “difficult” samples with high error, resulting in a better performance. A natural choice of weak learners for AdaBoost is realized as decision trees [20]. XGBoost is another boosting technique implementing gradient-boosted decision trees with advanced speed and performance [32]. Gradient boosting, which is a gradient descent method in function space capable of fitting non-parametric predictive models, has been empirically demonstrated to be accurate when applied to the tree models [19].

The parameters of AdaBoost and XGBoost were tuned experimentally: the best case was chosen among multiple cases with randomly selected parameters. For AdaBoost, the maximum depths of the base trees were set to 40, 50, and 41, respectively for the sphere, cylinder, and astigmatism axis. For the loss function, the exponential function was used for the sphere and astigmatism axis, whereas the linear function was used for the cylinder. For XGBoost, the maximum depths of the base trees were set to 34, 30, and 22, respectively for the sphere, cylinder, and astigmatism axis. The squared error was used as the loss function.

MLP, which is also called as an artificial neural network, comprises numerous layers of nodes and consists of an input layer, output layer, and multiple hidden layers in between [33, 34]. The strength of the connections between the interconnected nodes is expressed as weights, which are updated during the training process. The MLP models with the number of hidden layers of 1, 2, 4, 8, and 16 with 76 nodes were applied. The rectified linear unit activation function and the Adam optimizer were used. The learning rate was 0.001.

For each algorithm, three models for nomograms of sphere, cylinder, and astigmatism axis were trained, respectively. The process was performed using Scikit-learn [35]. We conducted a five-fold cross-validation. The overall data were divided into five groups randomly and the training and test process were repeated five times, where one group was used to test, whereas the other four groups were used to train. The test scores from the five repetitive processes were averaged. As scores to evaluate the performance, RMSE and accuracy with specific two thresholds were calculated. For the sphere and cylinder nomograms, the accuracy was calculated as the ratio of the output whose absolute difference between the ground truths was zero or smaller than 0.25 D. For the astigmatism axis, it was the ratio of the output whose absolute difference was 0 or smaller than 25°.

From the five trained models during five-fold cross-validation, the model with the lowest RMSE was selected as the best model. We correlated the ground truth, i.e., the nomograms of the expert ophthalmologist, and the output of the best models for all the data. We assessed the statistical significance of the correlations using the Mann–Whitney *U* test and the coefficient of determination, which is also called *r*^2^.

The feature importance of the best models was calculated using Breiman’s algorithm [36]. It was obtained by observing the increase in the mean absolute error when a specific feature was replaced with random noise. Subsequently, the obtained importance values were normalized between 0 and 1.

## Supplementary Information

**Additional file 1.** Names and statistical characteristics of the features and nomograms from the experts.

## Data Availability

The datasets used and/or analyzed during the current study are available from the corresponding author on reasonable request and with permission of the IRB.
