# Supplementary material for: Artificial intelligence-based nomogram for small-incision lenticule extraction
Source: Biomed Eng Online. 2021 Apr 23;20:38. doi: 10.1186/s12938-021-00867-7 (PMC8063457; doi:10.1186/s12938-021-00867-7)
Supplement: Supplementary file 1 — Additional file 1. Names and statistical characteristics of the features and nomograms from the experts. [file 12938_2021_867_MOESM1_ESM.docx]

**Supplementary element 1.** Names and statistical characteristics of the features and nomograms from the experts

| **Categorical features** | Percentage of the class |
| --- | --- |
| Surgeon | A: 40.48%, B: 59.46% |
| Right or Left | Right: 45.60%, Left: 54.40% |
| Patient demographic |  |
| Age | <25: 49.05%, 25–35: 42.85%, 35>: 8.10% |
| Gender | Male: 52.63%, Female: 47.37% |

| **Numeric features** | Mean ± SD | Range |
| --- | --- | --- |
| Preoperative manifest refraction |  |  |
| Spherical power* (D) | −3.74±1.47 | −8‒−0.5 |
| Cylindrical power* (D) | −0.81±0.65 | −3.25‒1 |
| Cylindrical axis* (°) | 128.79±70.02 | 1‒180 |
| Preoperative automated keratometry |  |  |
| Spherical power (D) | −3.95±1.54 | −8.25‒2.75 |
| Cylindrical power (D) | −1.06±0.70 | −4‒0 |
| Cylindrical axis (°) | 105.32±75.13 | 1‒180 |
| Flattest corneal curvature (D) | 42.51±1.39 | 38‒47.75 |
| Axis of flattest curvature* (°) | 101.08±82.34 | 1‒180 |
| Steepest corneal curvature (D) | 43.87±1.48 | 38.5‒49.25 |
| Axis of steepest curvature* (°) | 89.67±12.69 | 3‒180 |
| Corneal curvature cylinder (D) | −1.11±1.01 | −4.5‒3 |
| Corneal cylindrical axis (°) | 101.62±82.31 | 1‒180 |
| Pupil size in dark* (mm) | 6.70±0.69 | 4.2‒9.2 |
| Intraocular pressure* | 15.22±2.68 | 7‒25 |
| Corneal thickness* | 548.36±27.03 | 474‒639 |
| White-to-white* | 11.79±0.39 | 8.79‒13.77 |
| Optical zone* | 6.35±0.15 | 5.6‒6.7 |
| Pentacam |  |  |
| Anterior chamber depth* (mm) | 3.25±0.26 | 2.37‒5.95 |
| Anterior chamber angle* (°) | 41.31±5.02 | 24.3‒62.7 |
| Anterior chamber volume | 195.51±30.41 | 104‒340 |
| Keratometric power deviation* | 1.31±0.17 | 0.8‒2.2 |
| Corneal volume* | 63.50±3.25 | 54‒76.5 |
| X location at maximum corneal curvature* | −0.01±0.72 | −5.16‒4.49 |
| Y location at maximum corneal curvature* | 0.15±2.03 | −5.23‒5.04 |
| Maximum corneal curvature* (D) | 44.43±1.54 | 38.9‒50.9 |
| X location at thinnest cornea* | 0.05±0.51 | −1.43‒1.51 |
| Y location at thinnest cornea* | −0.44±0.22 | −1.47‒2.5 |
| Thinnest corneal thickness (µm) | 556.37±31.24 | 420‒651 |
| Corneal thickness at apex (µm) | 560.74±31.21 | 422‒655 |
| Pupil diameter (mm) | 2.88±0.51 | 1.7‒7.24 |
| X location at pupil center* | −0.02±0.13 | −0.56‒0.41 |
| Y location at pupil center* | 0.10±0.12 | −0.65‒0.63 |
| Corneal thickness at pupil center (µm) | 561.77±31.34 | 423‒656 |
| Corneal back eccentricity* | 0.24±0.23 | −0.52‒0.84 |
| Corneal back astigmatism* (D) | 0.42±0.15 | 0‒1.2 |
| Corneal back astigmatism axis* (°) | 89.85±83.85 | 0.1‒180 |
| Corneal back flattest curvature (D) | −6.13±0.24 | −7‒−5.3 |
| Corneal back steepest curvature (D) | −6.55±0.27 | −7.4‒−5.7 |
| Corneal back mean curvature* (D) | −6.33±0.24 | −7.1‒−5.5 |
| Corneal front eccentricity* | 0.42±0.23 | −1.29‒0.81 |
| Corneal front astigmatism (D) | 1.37±0.66 | 0‒3.8 |
| Corneal front astigmatism axis* (°) | 92.43±81.31 | 0.1‒180 |
| Corneal front flattest curvature* (D) | 42.37±1.48 | 33.9‒48.3 |
| Corneal front steepest curvature (D) | 43.73±1.63 | 34.3‒49.2 |
| Corneal front mean curvature (D) | 43.04±1.52 | 34.1‒48.1 |
| The selected features are marked with “*.” |  |  |

| **Postoperative outcomes** | Mean ± SD | Range |
| --- | --- | --- |
| Nomogram for sphere (D) | −4.18±1.53 | −8.7‒−0.9 |
| Nomogram for cylinder (D) | −0.90±0.69 | −3.5‒0 |
| Nomogram for cylindrical axis (°) | 121.46±73.48 | 1‒180 |
| Postoperative visual acuity (logMAR) | 1.11±0.11 | 0.9‒1.2 |
| Postoperative automated keratometry |  |  |
| Spherical power (D) | 0.09±0.42 | −4.5‒1.25 |
| Cylindrical power (D) | −0.45±0.25 | −1.75‒0 |
